# Impact of Chlorpyrifos on Cytopathological Indices in Mangrove Crab, *Episesarma tetragonum* (Fabricius)

**DOI:** 10.3390/vetsci10010053

**Published:** 2023-01-12

**Authors:** Rajesh Ravi, Maharajan Athisuyambulingam, Shanmugavel Kanagaraj, Nikola Tresnakova, Federica Impellitteri, Ganapiriya Viswambaran, Caterina Faggio

**Affiliations:** 1 PG & Research Department of Zoology, Khadir Mohideen College, Affiliated to Bharathidasan University, Tiruchirappalli, Thanjavur Dist, Adirampattinam 614701, India; 2 Research Institute of Fish Culture and Hydrobiology, South Bohemian Research Center of Aquaculture and Biodiversity of Hydrocenoses, Faculty of Fisheries and Protection of Waters, University of South Bohemia in Ceske Budejovice, Zatisi 728/II, 389 25 Vodnany, Czech Republic; 3 Department of Veterinary Sciences, University of Messina, Polo Universitario dell’Annunziata, 98168 Messina, Italy; 4 Department of Chemical, Biological, Pharmaceutical and Environmental Sciences, University of Messina, Viale Ferdinando Stagno, d’Alcontres 31, 98166 Messina, Italy

**Keywords:** insecticide, chlorpyrifos, crab, cytotoxicity, cytopathology

## Abstract

**Simple Summary:**

The purpose of this study was to see how chlorpyrifos, an organophosphate insecticide commonly found in an aquatic environment, affected the cytotoxicity of the mangrove crab, *Episesarma tetragonum*. For 7 and 28 days, specimens were exposed to 0.0294 and 0.0588 ppm chlorpyrifos, respectively. The gills, hepatopancreas, and muscles were examined for cytopathological effects. The findings indicate that chlorpyrifos causes cytopathological changes such as decreased epithelial lifting, edema, necrosis, secondary lamellae fusion, haemorrhage, haemocyte disappearance, muscle atrophy, and necrosis. Therefore, cytopathologic observation serves as a promising biomarker for ecotoxicological studies.

**Abstract:**

Chlorpyrifos is an organophosphate insecticide occurring in aquatic ecosystems. Due to exposure to xenobiotics, several harmful effects on aquatic organisms are noticed worldwide. Mangrove crabs are an ecologically important aquatic invertebrate species in food web interactions and in the mangrove ecosystem. Therefore, this study aimed to evaluate the cytotoxic effects of chlorpyrifos on the mangrove crab, *Episesarma tetragonum*. Crabs were exposed to 0.0294 and 0.0588 ppm of chlorpyrifos for 7 and 28 days. Cytopathologic effects on the gill, hepatopancreas, and muscle were investigated, and observations were compared with a control group. The results suggest that chlorpyrifos induces time- and concentration-dependent cytopathological alternations in the gill and exhibited epithelial lifting, oedema, necrosis, and a fusion of secondary lamellae and haemorrhage. The deceased hepatopancreas showed infiltration, a large lumen formation, and the disappearance of haemocytes, while the muscle tissue showed atrophy, necrosis, a wavy appearance, an accumulation of granular material between muscle fibres, and fragmentation in a mangrove crab. This study shows the great potential of cytopathological investigations, allows us to assess the sensitivity of various aquatic animal species to potentially dangerous compounds, and calculates safe concentrations with which to reduce pesticide use.

## 1. Introduction

Due to a strongly growing population and rising demand for grain, rice production has intensified worldwide. Paddy fields are temporarily flooded, and the pesticides used remain in the aquatic medium, resulting in a “water continuum” of water bodies. Simultaneously, a higher pesticide loss with drainage water and a continuous flow of water from one water body to another or from a field to another through a canal system. About half of the pesticides marketed in Asian markets are used for paddy fields [1]. Most pesticides such as dichlorodiphenyltrichloroethane (DDT), aldrin, dieldrin, endrin, mirex, heptachlor and hexachlorobenzene belong to a highly hazardous category and have deleterious consequences on human health and the environment [2]. In several emergent nations, which collectively account for a quarter of worldwide pesticide use cases, demand is rising even as regulatory approvals of chemical compounds used in pesticides decrease in the EU [3]. Farmers frequently lack reliable information about pests and how to control them, resulting in suboptimal spraying decisions. Farmers’ misunderstanding about the type of chemical, rate of application, and time of control is exacerbated by constantly changing pest complexes, a growing choice of insecticide treatments, and the lack of unique and specialized control advice for insect pests. These pest management methods kill insect pests and destroy their natural predators [4].

The second most popular organophosphorus agricultural pesticide in India is chlorpyrifos, known as O, O-diethyl-O (3,5,6-trichlor-2-pyridyl) phosphorothioate. According to the US Environmental Protection Agency, about 800 products containing chlorpyrifos are registered on the market. These commercial products are used for indoor pest control, structural pest control, pet collars, or for the protection of food crops, turf, and ornamental plants [5]. Due to its inexpensive cost, the availability of its formulations, and its effectiveness in small doses, chlorpyrifos is widely used in Bangladesh [6]. Most insecticides used for crops eventually find their way into surrounding bodies of water via canals, rain, and agricultural runoff. Pesticides have an important role in improving food quality and land production for the world’s expanding population, particularly in developing nations. Nonetheless, their presence in agricultural runoff poses a major danger to all aquatic ecosystem components [7].

In paddy fields, fish, frogs, rodents, crustaceans, aquatic insects and insect larvae, snails, worms, algae, and bacteria are vulnerable to chemical attacks. Because of the incorrect pesticide usage and application, poor rural communities, who rely on fish, shrimp, crab, and other organisms from paddy fields, are in grave danger. Widely used pesticides raise the possibility of environmental contamination and demonstrate their negative impacts on biodiversity, food security, and water resources [8,9,10,11,12,13]. The insecticide chlorpyrifos is found in agricultural, industrial, and home waste and is considered a primary source of aquatic contamination [14,15]. Nutrient recycling in paddy soils is also threatened by disrupting interactions between micro- and macro-organisms [16,17,18,19,20]. The mangrove habitat is particularly vulnerable to agricultural effluents, mainly because of its location at the river’s mouth. Natural and artificial forces put aquatic species in a wide range of physical and chemical circumstances, and, therefore, they are often under environmental stress.

Cytopathological indicators are crucial for monitoring and evaluating the toxicity of aquatic ecosystems [20,21,22,23,24,25,26]. Prolonged exposure to toxic agents often does not cause death directly; however, the structure and function of vital organs are affected, jeopardizing the individual population. Sometimes, the species and histological analysis of different organs may indicate the biological responses to an unfavourable situation among various biomarkers [27]. Moreover, it is generally known that the crustacean hepatopancreas plays a vital role in metabolism and the digestive system with basal function, such as food absorption, digestive enzyme regulation, and the storage of lipids, glycogen, and minerals during intermoult periods [28,29]. Additionally, it is the main detoxification organ, which is highly susceptible to ecotoxicological studies [25,30]. Details about ghost shrimp (*Palaemonetes argentines*) hepatopancreas morphology and histology with important tissular dynamics associated with the moulting cycle have been described previously by Sousa and Petriella [31].

Therefore, the aim of the present study was to evaluate the cytotoxic effects of 7 and 28 days of exposure to the insecticide chlorpyrifos on the gill, hepatopancreas, and muscle of mangrove crab *Episesarma tetragonum*.

## 2. Materials and Methods

### 2.1. Ethical Approval Declarations

Decapod crustaceans (such as lobsters and crabs) are exempt from current European legislation that protects animals used for scientific purposes because they are non-sentient and thus incapable of suffering; nevertheless, care and use of the experimental animals complied with local animal welfare laws, guidelines, and policies.

### 2.2. Test Animal Collection and Maintenance

Mangrove crabs (*Episesarma tetragonum*) with carapace sizes extending from 3.5 to 4 cm and weights 40–60 g were gathered from the mangrove region of Muthupettai, Thiruvarur Dist, Tamil Nadu (India). Crabs were moved to the Postgraduate and Research Department of Zoology Research laboratory, Adirampattinam, Tamil Nadu, India, for two weeks for acclimatization in a rectangular tank with 100 L aerated and purified estuary water kept at room temperature (27 ± 2 °C). The tanks were cleaned well before stocking and disinfected with 0.1% KMnO_4_. Before stocking, Crabs were examined for apparent pathogenic signs and treated with 0.1% KMnO_4_.

### 2.3. Tested Chemical

Organophosphorus (OP) is one of India’s most widely used pesticides, with applications in both agricultural and residential contexts. The commercial insecticide chlorpyrifos, with 50% of an active agent, was purchased from Shri Ram Agro Chemicals, Tamil Nadu, India. The stock solutions were diluted and dissolved with Milli-Q deionized water. Crabs were exposed to sublethal concentrations of 0.0294 and 0.0588 ppm, designed by 10 and 20% of the LC_50_ value for 96 h of acute toxicity experiments.

### 2.4. Test Procedure

Twenty healthy crabs (carapace size 3.7 to 4.7 cm, weight 45–70 g) were moved to the rectangular experimental tank after two weeks of acclimatization. The container was filled with 100 L of filtered, well-ventilated estuarine water added with different concentrations of chlorpyrifos. For the test and control concentrations, three replications were carried out. Crabs were given fresh chopped clams twice daily at 10:00 a.m. and 2:00 p.m., respectively; uneaten food was removed from the feeders to avoid organic contamination of the water. Crabs were fasted for 24 h before being sampled. Half of the experimental water was changed once in two days to keep the experimental pesticide concentration, and water quality (salinity: 13.5 ± 2.5 ppt, dissolved oxygen: 6.4 ± 1.0 mg/L, temperature: 24.5 ± 1.2 °C, and pH: 6.4 ± 1.4) was measured daily; additionally, ammonia nitrogen: 0.51 ± 0.45 mg/L and nitrite nitrogen: 0.48 ± 0.23 mg/L and nitrate nitrogen: 0.71 ± 0.19 mg/L were measured twice a week). All chemical parameters were determined to be within acceptable limits according to the standard procedure followed by the American Public Health Association [32]. Mortality and behaviour were observed every day in each experimental group. Five crabs from each aquarium were sampled at 0, 7, and 28 days post-exposure.

### 2.5. Cytological Analysis

At the sampling times, crabs were killed by piercing both ganglia from the crab’s underside with a pointed spike and rapidly destroying both nerve centres. The gill, hepatopancreas, and muscle tissues were then dissected and fixed in 10% buffered formalin for 24 h before being dried in a graded ethanol series and embedded in paraffin. Tissue sections (5 mm thick) were stained with haematoxylin and eosin before being examined using a Nikon bright-field transmission microscope coupled with Koehler illumination and automated exposure equipment.

## 3. Results

### 3.1. Cytology of Gill

**Figure 1:**
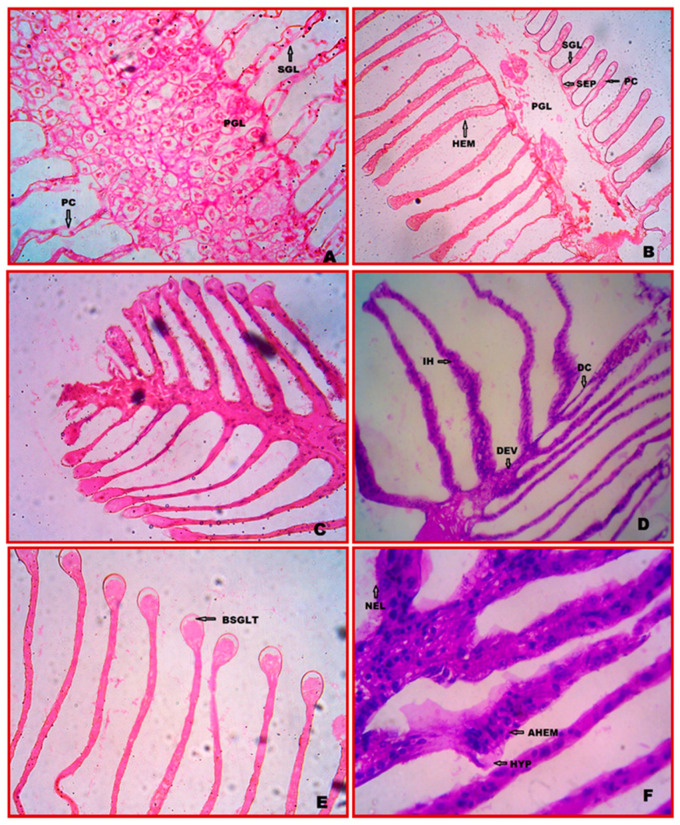
Gill cytopathological alterations in *E. tetragonum*: A paraffin slice stained with hematoxylin and eosin under the light microscope (40×), (**A**,**B**)—control (**C**)—after 7 days of exposure to chlorpyrifos at a dosage of 0.0294 ppm, (**D**)—after 28 days of exposure to chlorpyrifos at a dosage of 0.0294 ppm, (**E**)—after 7 days of exposure to 0.0588 ppm chlorpyrifos concentration, (**F**)—after 28 days of exposure to 0.0588 ppm chlorpyrifos concentration. Abbreviations used: SGL—secondary gill lamellae, PCs—pillar cells, HEM—haemocytes, PGL—primary gill lamellae, SEP—septum, DC—detached cuticle, IH—infiltration of haemocytes, DEV—damaged efferent vessel, BSGLT—bulging of secondary gill lamellae tip, NEL—necrosis, AHEM—aggregation of haemocytes, HYP—hypertrophy.

The gill tissue of *E. tetragonum* is made up of lamellae, or large, flattened plates that are placed serially in pairs along a control gill stem. The major gill lamellae are the core axis of gill tissue and are further divided into secondary gill lamellae or filaments. The whole exterior surface of the control gill is covered by a thin cuticle layer. Under the cuticle is a continuous layer of epithelial cells. Pillar cells connect the lamellae at unequal intervals. The lamella’s distal portion is enlarged. The gill stem’s lining comprises epithelial cells from the lamellae, while the gill stem’s main support comprises massive connective tissue cells (Figure 1A,B).

### 3.2. Cytopathology of Gill

The crabs treated with chlorpyrifos concentrations showed several cytological changes after 7 and 28 days of exposure. The gills showed cytoplasmic vacuolization in the gill stem. The primary and secondary gill lamellae were ruptured, and connective tissue cells in the stem, efferent vessels, and detached cuticles were damaged. The bulged secondary gill lamellae (BSGLT) and the pillar cells (PCs) are damaged. In the absence of the PCs, these diseases collapse the whole lamellae (0.0294 ppm (1/10th of LC_50_ value for 96 h) (Figure 1C,D). The secondary gill lamellae are necrosis (NEC) in the higher chlorpyrifos concentration. Aggregation (AHEM) and infiltration (IH) of haemocytes in the gill lamellae were observed, and hypertrophy was present after 28 days of exposure at 0.0588 ppm (1/20th LC_50_ value for 96 h) (Figure 1E,F).

### 3.3. Cytology of Hepatopancreas

The hepatopancreas segment revealed numerous connective tissue-supported elongated tubules that were pink from haematoxylin and eosin staining. Each tubule has an inner layer of epithelial cells and a thin outer cuticle. The centre chamber of each lumen, which was covered by a thin epidermal layer, varied in size and shape within each tubule.

**Figure 2:**
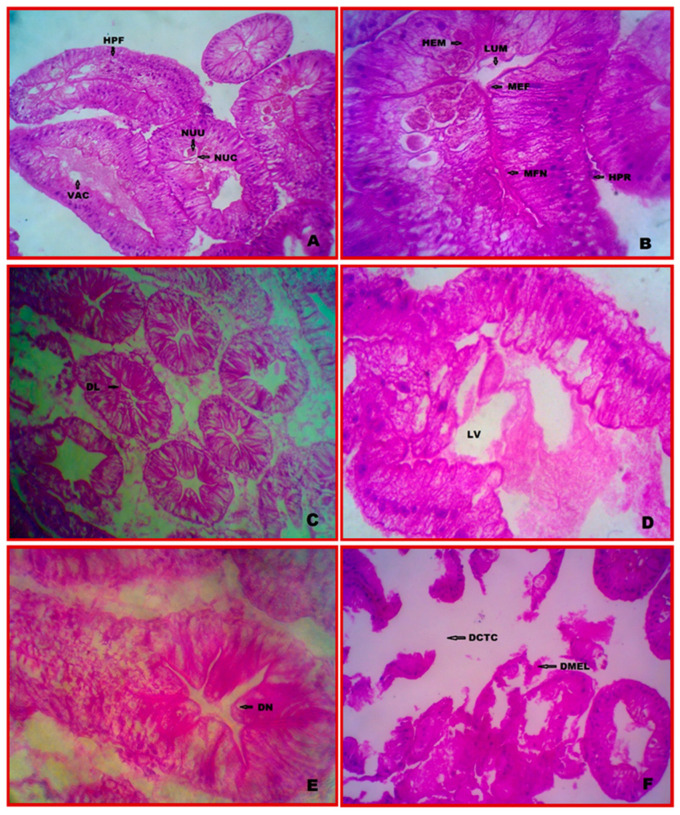
Cytopathological alterations of hepatopancreas in *E. tetragonum*. A paraffin slice stained with hematoxylin and eosin (40×) under the light microscope: (**A**,**B**)—control, (**C**)—after 7 days of exposure to chlorpyrifos at a dosage of 0.0294 ppm, (**D**)—after 28 days of exposure to chlorpyrifos at a dosage of 0.0294 ppm, (**E**)—after 7 days of exposure to chlorpyrifos at 0.0588 ppm, (**F**)—after 28 days of exposure to chlorpyrifos at 0.0588 ppm. Abbreviations used: HPF—F-cells, NUC—nucleus, NUU—nucleolus, VAC—vacuole, LUM—lumen, MEF—myoepithelial cells associated fibres, MFN—myoepithelial cells prominent nuclei, HPR—R cells, DL—distended lumen, LV—large vacuole, DCTC—damaged connective tissue layer, DN—disappearance of the nucleus, DMEL—damaged myoepithelial layer.

According to histological analysis, the tubules were comprised of an epithelium consisting of four cell types B (blister-like), E (embryonic), F (fibrillar), and R (receptor) (resorptive). Undifferentiated polyhedral cells composed the E-cells. They proliferated close to the distal tip of the tubules, where they were concentrated and showed a high nucleocytoplasmic ratio. The basally positioned nuclei of the F-cells’ tall columnar epithelial cells. They were tall columnar epithelial cells with a well-developed and rough endoplasmic reticulum that provided the appearance of being striated. They serve as secretaries in the mesiodistal and medioproximal areas of the tubules. The most significant hepatopancreatic cell type is located primarily in the proximal regions of the tubules, with compressed basal nuclei and a single massive vacuole. The R-cells’ cytoplasm was multi-vacuolated. They were found in the proximal and mesiodistal regions of the tubules (Figure 2A,B).

### 3.4. Cytopathology of Hepatopancreas

In treatment with a lower concentration (10% LC_50_, 0.0294 ppm) of chlorpyrifos, several changes were observed during the 7- and 28-day period. Large vacuoles, distended lumen, and an extensive general degeneration of the tubular and intratubular tissues have appeared most in hepatopancreas cells. In addition, tissue vacuolation, a total loss of tubular structures, and necrosis were detected (Figure 2C,D). In treatment with a higher concentration (0.0588 ppm) of chlorpyrifos, the disappearances of the nucleus (DN) and the connective tissue layer damage (DCTC) were observed after 28 days, along with elongated haemocytes, thickening of the basal lamina, a reduction in the cell height bulging of the myoepithelial layer, a damaged myoepithelial layer (DMEL), and bulging of the myoepithelial layer. In a severe case, more haemocytes surround the damaged capillaries (Figure 2E,F).

### 3.5. Cytology of Muscle

**Figure 3:**
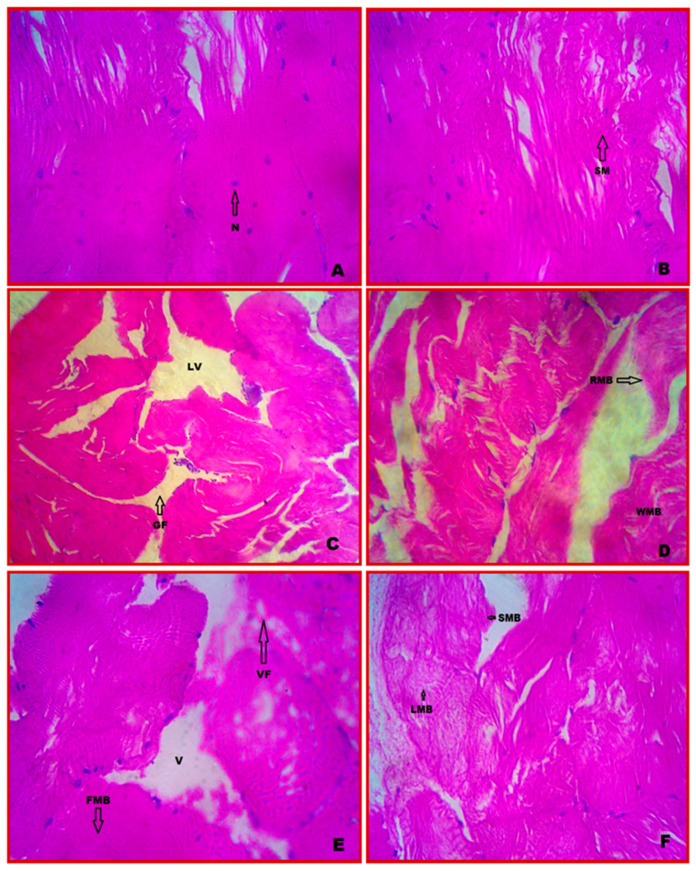
Cytopathological alterations of muscle in *E. tetragonum*. A paraffin slice stained with hematoxylin and eosin (40×) under the light microscope: (**A**,**B**)—control, (**C**)—after 7 days of exposure to chlorpyrifos at a dosage of 0.0294 ppm, (**D**)—after 28 days of exposure to chlorpyrifos at a dosage of 0.0294 ppm, (**E**)—after 7 days of exposure to chlorpyrifos at 0.0588 ppm, (**F**)—after 28 days of exposure to 0.0588 ppm chlorpyrifos concentration. Abbreviations used: SM—striated muscle, N—nuclei, LV—large vacuole, GF—gap formation, RMB—rupture of muscle bundle, WMB—wavy of muscle bundle, VF—vacuole formation, V—vacuole, FMB—fusion of muscle bundle, SMB—shortening of muscle bundle, LMB—loosen of muscle bundle.

Muscle cells in the *E. tetragonum* control muscle tissue had contractile filaments that moved and altered the size of the cell. Protein is present in the muscle tissue generated from mesoderm, and myosin filament (thread-like) creates multinucleate cells that come together to form fibres known as myofibrils (Figure 3A). Normal myotomes with regularly spaced muscle bundles were observed in the muscle photomicrograph, as well as a fascicular distribution of myofilaments with marginalised epimysium connecting to connective tissue and tendon at the ends of the smooth muscle. The striated muscle fibres were quite densely packed. The nuclei were positioned at the muscle bundles’ margins (Figure 3B).

### 3.6. Cytopathology of Muscle

After 7 and 28 days of exposure to chlorpyrifos, the muscle tissue showed disintegration of the epidermis with vacuoles, gaps (GFs) between the muscle bundles, large vacuoles (LVs), muscle bundle rupture (RMB), muscle bundle wavy (WMB), marked thickening and separation of the muscle bundle, and pronounced intramuscular oedema with minor dystrophy (Figure 3C,D). At the highest concentration (0.0588 ppm) and after 28 days of exposure, the muscle bundles were totally destroyed with discontinuity of the striations and the complete disappearance of the nuclei and formation of vacuoles. Fusion of muscle bundles (FMBs), loosening of muscle bundles (LMBs), and the shortening of muscle bundles were observed in certain regions of muscle tissue (Figure 3E,F).

Compared to the control crab group, the exposed crab groups exhibit greater lesion severity, as seen in Table 1.

## 4. Discussion

The cytotoxic effects of chlorpyrifos on the tissues of mangrove crabs were thoroughly investigated. The examination of the cytology of a given tissue is an important diagnostic technique for observing the histological influence of a pollutant. To identify pollution exposure, tissue histology can be used to evaluate the degree of contamination, particularly for sublethal and chronic impacts [33]. Due to their location and huge external area, fish gills are exposed to contaminants in water [13]. Pesticides have similar effects on fish gills, which are responsible for several functions such as respiration, digesting, osmoregulation, and excretion. According to Cengiz and Unlu [33], pesticides have similar effects on fish gills, which are responsible for several functions such as respiration, digesting, osmoregulation, and excretion. The histological changes may manifest in sick tissues [34]. In a recent study, it was found that an increase in exposure period, though exposed to a lower concentration, leads to increases in damage to the tissues of the mangrove crab.

The gills and the hepatopancreas are two of the most important metabolic organs in crustaceans, performing a wide range of functions. These crustaceans’ highly plastic organs allow them to adapt to various environmental changes. The gills, in particular, are the first line of defence of aquatic organisms’ tissues against toxic agents in their environment and are thus expected to be severely affected during exposure. This organ’s larger surface area is a biological barrier between the polluted medium and the inner compartment [35]. The phyllo brachiate gills of the crab *E. tetragonum* have two rows of gill lamellae on either side of a central axis (Figure 1). Regarding the gills’ anatomy, this study agreed with earlier findings on several crabs [36,37]. The experimental crab gills histological sections revealed vacuolization in the gill stem, gill lamellae rupture, damaged connective tissue cells in the stem, and congestion of haemoglobin-producing cells. The two-gill lamellae were ruptured by a thin connective fluid band (Figure 1). Similar results were observed in structural alterations in the giant freshwater prawn *Macrobrachium rosenbergii* when subjected to waterborne copper, including swelling and fusing of the lamellae, aberrant gill tips, and necrotic lamellae [38]. In this regard, the swelling and lifting of the lamellar epithelium observed in both studies and changes in the gill lamellae may reflect a physiological adaptation to the stress caused by pollutant exposure. On the other hand, the loss of cell ions could aggravate the damage of gill function and result in lower oxygen uptake and, in the end, asphyxia. Earlier, after nickel exposure, Kurian and Radhakrishnan (2002) noticed comparable alterations in the gills of the field crab, *Paratelphusa hydrodromus.*

Gills must be in intimate contact with the external medium to perform gaseous exchanges and ionic regulation. Because they are the main targets of pollution, gills are living organisms. These locations are related to dangerous substances with different charges, mechanical reactions, and poisonous effects on the organism that improve the normal function of the organ. After being exposed for 28 days, the crab’s gills were inspected, and it was found that there was the lifting of the epithelium, oedema, epithelial necrosis, fusion of secondary gill lamellae, and haemorrhaging at the primary gill lamellae. The irritants’ immediate adverse effects include epithelial necrosis and gill epithelium rupture. Severe damage in terms of necrosis and rupture of the branchial epithelium causes hypoxia and respiratory failure. Excessive mucus secretion is the animal’s defence mechanism. Moreover, the lifting of the epithelium, a damaged epithelium, lamellar fusion, and club-shaped lamellae decrease the sensitive gill surface area and may be protective [39].

The effects of diazinon and sodium dodecyl sulphate cause severe necrotic lamellae in the gill tissues of *Rutilus rutilus*, according to research by Katuli et al. [40]. Similar effects of diazinon exposure included oedema, epithelial lifting, secondary lamellae curling, secondary lamellae shortening, and lamellar fusion in the gills of *Scatophagus argus* [41]. The reported histological results of hyperplasia, necrosis, and lamellar fusion, as well as damaged epithelium in the exposed crustaceans because of sub-lethal doses of chlorpyrifos, are connected with alterations in the gill surface and higher mucus production. Under the effects of stress, pesticides, and chlorpyrifos, alterations in the construction of the gill would affect its ability to diffuse oxygen, leading to hypoxic conditions. As a result, breathing becomes difficult for the crab in its estuary habitat. The present work hypothesizes that chlorpyrifos’ deadly effects are caused by damage to gas exchange mechanisms, which impacts the diseases seen in the gills.

Initially, only the digestive gland in crustaceans was considered; however, the hepatopancreas is now thought to play a role in intermediate metabolism and serve as a significant depot for fat bodies, such as the adipose tissue found in the livers of vertebrates, which is a sensitive organ and liable to be damaged by waterborne pollutants [42]. In fact, the hepatopancreas is not only a digestive organ with absorption, digesting, storage, and secretion capabilities. It is also an essential site of biotransformation and detoxification which plays a role in hazardous agent defence [43]. It is a prominent location for storing minerals, lipids, proteins, and carbs. Crustacean hepatopancreas is primarily made of branching tubules. Rest zellen cells (R-cells) are one type of present-day cells with absorptive capabilities supported by cytoplasmic nutrition stores.

Through the haemolymph, R-cells transport nutrients to other organs and then mobilize the resources to supply energy to the rest of the body. Heavy metals and other lipophilic substances are detoxified by these cells by accumulating in the cytoplasm in a soluble form, followed by excretion [31]. In the hepatopancreas, toxic substances have effects such as necrosis of the tubules, inflated F-cells, infiltration of cells in the interstitial sinus, aberrant tubule lumen, and dissociation of necrotic cells from the basal laminae. The necrotic hepatopancreatic tubules in the studied crabs imply that the animals’ hepatopancreas underwent deformation, disintegration, and cell death. Thus, chlorpyrifos’ exposure disrupts the normal function of the examined organs, causing serious harm to the organism. The gills and other organs are also harmed, such as the hepatopancreas. Since other bodily parts need the energy to recuperate from adverse effects, the R-cell is essential in transporting energetic resources. Deficits in hepatopancreatic function may result from the interaction of all these effects [44,45,46,47]. The current examination of *E. tetragonum* found haemocyte aggregation and hemosinus enlargement in the hepatopancreas following the test pesticide exposure, which is consistent with the previous reports. Because haemocytes are the most significant type of cellular protection in crabs, the anomalous infiltration of haemocytes seen in the hepatopancreas of mangrove crabs shows that the cellular/host defence mechanism was in operation to neutralize tissue damage produced by chlorpyrifos [43].

Infiltration of haemocyte-damaged epithelial cells, the formation of pyknotic nuclei, cytolysis, and the encapsulation of necrotic tissues were among the structural alterations caused by chlorpyrifos in the hepatopancreas of crabs. Previous research on the hepatopancreas at several different levels, including the structure, development, physiology, metabolism, and biochemistry, revealed that the hepatopancreas performs various tasks, including absorption, digesting, storage, and secretion [48,49]. Similarly, research on insecticides and fungicides’ impact on decapods has been conducted [47]. Because the hepatopancreas is the core of the storage organ for metabolism and decontamination, the observed histopathological changes in this organ could be due to pesticide accumulation.

Pollutants immediately affect the muscle epidermis, the major exposure site. The presence of pigmented cells is a common hallmark of chronic inflammatory responses. The findings of this study matched those of Tehrani et al. [50] in the muscular tissues of *Artemia urmaiana* in response to carbamates pesticide-induced degeneration and Zenker’s necrosis of muscle fibre with haemorrhages and RBC-like cells. The exposure of hexachlorocyclohexane to *Labeo rohita* induced the separation of muscle bundles and intracellular oedema in the muscle tissues [51].

Moreover, Fatma [52] found degeneration of muscle bundles in the muscle tissues of *Tilapia zillii* and *Solea vulgaris* subjected to heavy metal and aggregations of inflammatory cells and isolated areas of necrosis. On exposure to dimethoate, similar observations were made in the muscular tissues of *Oreochromis mossambicus* [53,54], and histopathological changes were found in the muscle tissues of *Heteropneustes fossilis* subjected to contaminated river water. After sublethal exposure to chlorpyrifos in crab, *E. tetragonum*, the present study discovered oocyte membrane rupturing, vacuolization in peripheral oocytes, and abnormalities in the supporting connective tissue.

Following exposure to sub-lethal amounts of chlorpyrifos, various cytopathological changes in the muscles of *E. tetragonum* were observed. Muscle degeneration, necrosis of muscular fibres with haemorrhages, and muscle bundle shortening are pathological signs. The structural changes in muscle tissue, such as atrophy, necrosis, wavy appearance, granular material between muscle fibres, fragmentation, loss of muscle structure, and the formation of basophilic deposits of muscle fibres, were caused by sub-lethal exposure to crabs.

Because muscle tissue is the primary exposure location, contaminants immediately affect the muscular epidermis. The presence of pigmented cells is a common hallmark of chronic inflammatory responses. The findings of this study matched those of Tehrani et al. [50] in the muscular tissues of *Artemia urmaiana* in response to carbamates pesticide-induced degeneration. The reports mentioned before support these current findings.

## 5. Conclusions

Biomarker changes in juvenile crabs depended on tissue type and exposure time. This study discovered links between the responses of cytological indicators to chlorpyrifos-contaminated water. Due to the inefficient and overuse of pesticides, such as the insecticide chlorpyrifos, by local farmers in paddy fields to gain higher production in Asia, the importance of monitoring its potential risks on non-target aquatic organisms is still needed. These findings have significant implications for the aquacultural management of this crab species and highlight the need for further investigation. As a result, this work can raise awareness among local farmers, assess the sensitivity of various aquatic animal species to effluent potency using LC_50_ values, and calculate safe concentrations so that pesticide use is reduced and biopesticides are used instead.

## Figures and Tables

**Table 1:** Cytopathologic observations of mangrove crab, *E. tetragonum*, after the duration of 7-d and 28-d to sublethal concentration of insecticide Chlorpyrifos (0.0294 and 0.0588 ppm). Legend: –, none (0%); +, mild (<10%); ++, moderate (10 to 50%); +++, severe (>50%).

Cytopathology		Experimental Groups				
Tissue	Indices	Control	0.0294 ppm		0.0588 ppm	
			7-d	28-d	7-d	28-d
Gills	Detached cuticle (DC)	–	–	++	–	–
	Necrosis (NEC)	–	–	–	–	+++
	Infiltration of haemocytes (IH)	–	–	++	–	–
	Damaged efferent vessel (DEV)	–	+	++	++	–
	Bulging of secondary gill lamellae tip (BSGLT)	–	–	–	–	–
	Aggregation of haemocytes (AHEM)	–	–	–	–	++
	Hypertrophy (HYP)	–	–	–	–	++
Hepatopancreas	Distended lumen (DL)	–	+	–	++	–
	Large vacuole (LV)	–	+	++	–	–
	Damaged connective tissue layer (DCTL)	–	–	–	–	++
	The disappearance of the nucleus (DN)	–	–	++	++	+++
	Damaged myoepithelial layer (DMTL)	–	–	–	+	+++
Muscle	Large vacuole (LV)	–	++	–	–	–
	Gap formation (GF)	–	++	++	–	–
	Rupture of muscle bundle (RMB)	–	–	++	–	–
	Wavy muscle bundle (WMB)	–	–	–	–	–
	Vacuole formation (VF)	–	–	–	++	–
	Fusion of muscle bundle (FMB)	–	–	–	+++	+++
	Shortening of muscle bundle (SMB)	–	–	–	–	+++
	Loosen of muscle bundle (LMB)	–	–	–	–	–

## Data Availability

Data and detailed methodology information are available from the first author under reasonable request.
